# Gene delivery using AAV8 *in vivo* for disease stabilization in a bimodal gene therapy approach for the treatment of ADA-deficient SCID

**DOI:** 10.1016/j.omtm.2021.02.007

**Published:** 2021-02-15

**Authors:** Denise A. Carbonaro-Sarracino, Krista Chun, Danielle N. Clark, Michael L. Kaufman, Xiangyang Jin, Xiaoyan Wang, Donald B. Kohn

**Affiliations:** 1 Department of Microbiology, Immunology, and Molecular Genetics, University of California, Los Angeles, Los Angeles, CA 90095, USA; 2 Department of General Internal Medicine and Health Services Research, University of California, Los Angeles, Los Angeles, CA 90095, USA; 3 Department of Pediatrics, David Geffen School of Medicine, University of California, Los Angeles, Los Angeles, CA 90095, USA; 4 The Eli & Edythe Broad Center for Stem Cells and Regenerative Medicine, University of California, Los Angeles, CA 90095, USA

**Keywords:** AAV vector, gene therapy, lentiviral vector, ADA-SCID, ADA-deficiency, inborn errors of metabolism, hematopoietic stem cells, severe combined immunodeficiency

## Abstract

Adenosine deaminase (ADA) deficiency is an inborn error of metabolism affecting multiple systems and causing severe combined immunodeficiency. We tested intravenous administration of recombinant adeno-associated virus (AAV) 2/8-ADA vector in ADA-deficient neonate and adult mice or as part of a bimodal approach comprised of rAAV treatment at birth followed by infusion of lentiviral vector (LV)-modified lineage-depleted bone marrow cells at 8 weeks. ADA^−/−^ mice treated with rAAV and enzyme replacement therapy (ERT) for 30 days were rescued from the lethal pulmonary insufficiency, surviving out to 180 days without further treatment. rAAV vector copy number (VCN) was highest in liver, lung, and heart and was associated with near-normal ADA activity and thymocyte development. In the bimodal approach, rAAV-mediated *ADA* expression supported survival during the 4 weeks before infusion of the LV-modified bone marrow cells and during the engraftment period. Conditioning prior to infusion may have resulted in the replacement of rAAV marked cells in marrow and liver, with LV VCN 100- to 1,000-fold higher in hematopoietic tissue compared with rAAV VCN, and was associated with immune cell reconstitution. In conclusion, a bimodal approach may be an alternative for patients without reliable access to ERT before receiving a stem cell transplant or gene therapy.

## Graphical Abstract

**Figure:**
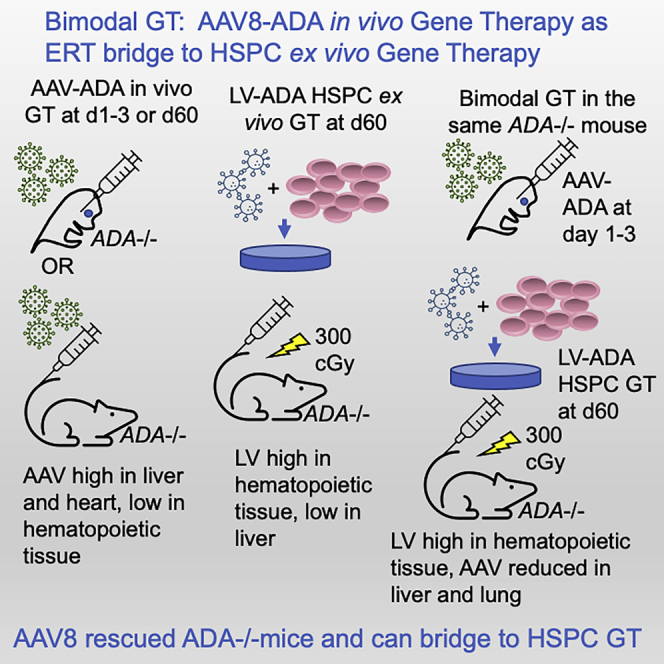

## Teaser

ADA-deficient mice were treated with rAAV8-ADA alone or as a bimodal gene therapy, with rAAV given at birth and LV-mediated *ex vivo* HSPC gene therapy after post-natal day 60. Treated mice survived 180 days with either approach, demonstrating rAAV-ADA may be an alternative for patients without reliable access to enzyme replacement.

## Introduction

Adenosine deaminase (ADA) deficiency is an inborn error of metabolism caused by a mutation in the *ADA* gene and results in a severe combined immunodeficiency (ADA-SCID) and other system manifestations.^1^^,^^2^ ADA is a critical enzyme in the purine salvage pathway and is responsible for catalyzing the deamination of metabolites, deoxyadenosine and adenosine, to deoxyinosine and inosine, respectively.^2^ Without active ADA, these substrates accumulate and are converted to deoxyadenosine triphosphate (dATP), which at high concentrations is particularly cytotoxic to lymphocytes, resulting in SCID.^3^ Affected patients have an extremely compromised immune system that cannot fight off infections effectively that often results in early mortality when untreated.^4^ Current standards of care include enzyme replacement therapy (ERT) with pegylated ADA (PEG-ADA), hematopoietic stem cell transplantation (HSCT), and autologous transplant of genetically modified hematopoietic stem cells *ex vivo* gene therapy (HSC *ex vivo* GT).^5^

ERT, either supplied as bovine PEG ADA (Adagen; Leadiant Biosciences, Gaithersburg, MD, USA) or, more recently, as recombinant bovine PEG-ADA (Revcovi; Leadiant Biosciences, Gaithersburg, MD, USA), is used for the rapid reduction of metabolites and can be lifesaving. However, as a long-term treatment, ERT has been associated with waning immunological function and reduced survival.^6^^,^^7^ HSCT can be a definitive treatment, especially for those patients with a matched related donor. However, for many patients, a fully matched related donor is often unavailable, and a less suitable donor may be used. In ADA-deficient patients, donor suitability is directly correlated to survival, and the probability of surviving transplant with mismatched donor cells decreases to less than 60%.^8^ Due to insufficiencies with HSCT and PEG-ADA ERT, HSC *ex vivo* GT has been developed and is an approved treatment modality in the European Union (EU) (Strimvelis; Orchard Therapeutics) when a histocompatibility leukocyte antigen (HLA)-matched sibling donor is not available.^9^

It is generally recognized that most patients in the United States and in the EU are stabilized with ERT prior to treatment with a definitive therapeutic such as HSCT or HSC *ex vivo* GT.^5^ Stabilizing with ERT results in systemic detoxification and initiation of immune reconstitution and can be lifesaving.^5^ Typically with HSCT and with the use of Strimvelis, ERT is normally discontinued immediately prior to transplant.^8^^,^^10^ However, studies in *ADA*^*−/−*^ mice demonstrated that engraftment was greatly improved, especially in the thymus, when mice remained on ERT through the initial engraftment period.^11^ Additionally, ERT for 30 days after lentiviral vector (LV)-mediated HSC *ex vivo* GT has been translated to the clinic and has not shown deleterious effects of ERT, such as negation of the selective pressure on corrected cells.^5^

In these proof-of-concept studies, recombinant adeno-associated virus (rAAV) 2/8 carrying a normal human *ADA* gene (cDNA) was delivered intravenously into *ADA*^*−/−*^ mice to test the feasibility and efficacy of two modalities of treatment: (1) as a sole rAAV *in vivo* GT in newborn and adult *ADA*^*−/−*^ mice, and (2) as a bimodal GT combining the use of rAAV2/8 with a subsequent treatment with LV-mediated HSC *ex vivo* GT. Conceptually, this type of therapy would be most useful for those patients living in remote areas, where shipment of a single dry ice package is often easier than repeated shipments of a live enzyme necessitating precise refrigeration temperatures for the entire journey. We hypothesized that the two-prong approach of bimodal GT would provide initial stabilization of the disease phenotype and would not be a detriment to efficacy or safety with a subsequent HSC *ex vivo* GT. The results of these experiments demonstrate that *ADA*^*−/−*^ mice can be rescued with rAAV2/8 *in vivo* GT as the sole source of ADA ERT, and that bimodal GT for ADA-deficient SCID is feasible, safe, and efficacious.

## Results

In mice, knockout (KO) of the *ADA* gene is embryonic lethal^12^, ^13^, ^14^ but is rescued with a two-stage genetic engineering strategy in which an *ADA* mini-gene trophoblast expression cassette was knocked in for placental ADA expression during the gestational period.^15^ Once born, the *ADA*^*−/−*^ mice are completely ADA deficient, and accumulation of adenosine and deoxyadenosine results in multisystem abnormalities, including death at post-natal days (P) 19–20 from pulmonary insufficiency.^15^

The first dose-response experiments were performed in the *ADA* KO mouse model on the FVB/129 strain background. The rAAV was engineered with an AAV2 backbone and an AAV8 serotype, and titer ranged between between 2.4 × 10e12 and 4.8 × 10e12 genome copies (gc)/mL. Litters of neonatal *ADA*^−/−^ and *ADA*^+/−^ mice were treated with 1.0 × 10e10, 1.0 × 10e11, or 1.0 × 10e12 gc/kg of either γ retroviral MND U3 promoter/enhancer (MND)-ADA rAAV8 or elongation factor-α gene “short” promoter (EFS)-ADA rAAV8, administered intravenously via the temporal facial vein. It should be noted that the *ADA*^+/−^ mice do not display a disease phenotype and were used as controls.^16^ Mice were maintained on ERT for 30 days after rAAV administration to avoid large numbers of intercurrent deaths before the rAAV derived ADA expression had achieved maximum output.

**Figure 1:**
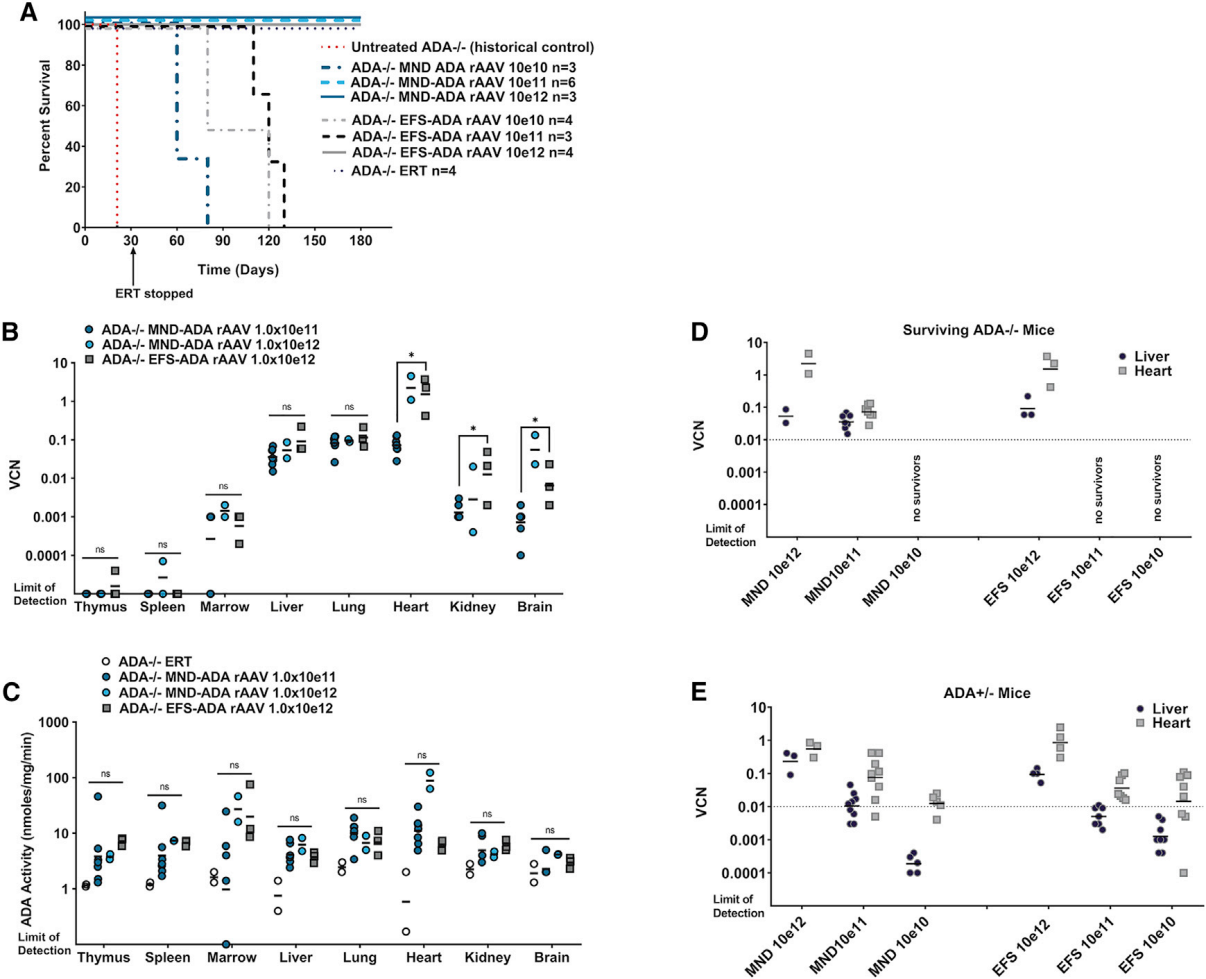
Dose-response analysis after systemic administration of rAAV *in vivo* GT in *ADA*^−/−^ and *ADA*^+/−^ neonates (A) Survival after treatment with intravenous injection of rAAV *in vivo* GT; survival was tracked to day +180, at which time the animals were euthanized for analysis. Treated mice were compared with historic controls of untreated mice that died by the third week of life. Survivorship was subjected to Kaplan-Meier analysis. (B) Tissue rAAV VCN. (C) Tissue ADA enzyme activity. (D and E) Dose response (VCN) in liver and heart of rAAV in *ADA*^−/−^ mice (D) and in similarly treated *ADA*^+/−^ littermates (E). Dotted line was drawn just below the lowest marked tissue in the surviving *ADA*^−/−^ mice to indicate the putative threshold for survival in *ADA*^−/−^ mice treated with rAAV. (B–E) Geometric means are plotted; Wilcoxon rank-sum test: ∗p < 0.05. ns, no significant difference.

All pups tolerated the injection of the rAAV; however, survival was poor in mice treated with the lower doses of either vector, with no *ADA*^*−/−*^ mice surviving when treated with less than 1.0 × 10e11 gc/kg of either vector (Figure 1A). Compared with historic controls of untreated *ADA*^*−/−*^ mice, the probability of survival was significantly increased after treatment with the MND-ADA rAAV8 at the highest two doses (1 × 10e11, p = 0.003; 1 × 10e12, p = 0.01) and EFS-ADA rAAV8 at the highest dose (1× 10e12, p = 0.008). Furthermore, there was a significant dose-response relationship with survival after treatment with MND-ADA rAAV8 (p = 0.002).

At 6 months, the surviving *ADA*^*−/−*^ mice were euthanized, and tissues were harvested for analysis. In some groups, it was not possible to discern if there was significant dose response with vector copy number (VCN) or ADA activity because so few animals survived when treated with the lower doses of rAAV (Figures 1B and 1C). rAAV vector marking, as determined by tissue, VCN, was highest in the heart (VCN range, 0.130–4.52), liver (VCN range, 0.033–0.219), and lung (VCN range, 0.067–0.212) and was uniformly low in the hematopoietic tissues (VCN range detection limit at 0.00001–0.002). Tissue ADA enzyme activity in ADA^−/−^ mice treated with rAAV. did not have a clear relationship to the VCN detected in the tissue. It is not clear if this was due to a limit on the capacity of some tissue to produce high levels active enzyme or if some of the rAAVs genomes were altered to limit transgene expression. In ADA^−/−^ mice treated with ERT, PEG-ADA remains extracellular, and tissue activity detected is at the limit of detection of the ADA assay and is considered negative.

In the surviving mice treated with rAAV *in vivo* GT, it was noted that all surviving *ADA*^−/−^ mice had a liver VCN at or above 0.01 (Figure 1D). To more fully understand the efficacious dose range of rAAV in ADA-deficient mice, we analyzed VCN in heart and liver from similarly treated *ADA*^+/−^ heterozygous littermates to determine if there was a dose response across all doses tested, especially in those lower doses that did not support survival of similarly treated *ADA*^−/−^ mice. Indeed, there was a significant dose response with VCN detected in heart (p < 0.0001) and liver (p < 0.0001) in *ADA*^+/−^ mice treated with MND-ADA rAAV across all doses tested (Figure 1E). Additionally, in *ADA*^+/−^ mice treated with lower doses in which no *ADA*^−/−^ mice survived, liver VCN was lower than 0.01, suggesting that there is a marking threshold associated with adequate ADA expression to support survival of *ADA*^*−/−*^ mice.

**Figure 2:**
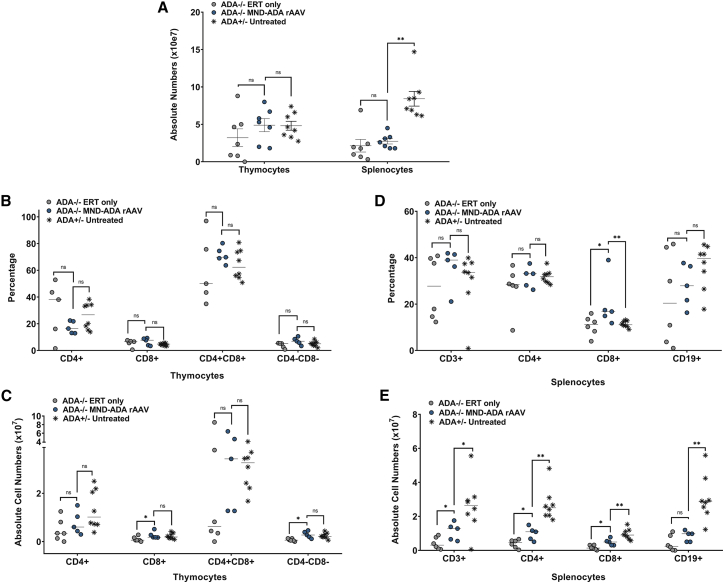
Immunophenotype and absolute cell counts after 6 months post-rAAV *in vivo* GT in *ADA*^−/−^ and *ADA*^+/−^ neonates Comparisons were made with untreated *ADA*^−/−^ mice euthanized at 16 days of age prior to serious decline and death, as well as with age-matched *ADA*^−/−^ mice maintained on ERT and untreated *ADA*^+/−^ mice. (A) Total lymphoid organ cell counts. (B) Thymocyte subpopulations (percentage): single-positive CD4^+^, single-positive CD8^+^, double-positive CD4^+^CD8^+^, and double-negative CD4^−^CD8^−^. (C) Absolute thymocyte counts. (D) Splenocyte subpopulations: CD4^+^ helper T cells, CD8^+^ cytotoxic T cells, CD3^+^ T cells, and CD19^+^ B cells. (E) Absolute splenocyte counts. (A–E) Mean ± SEM are plotted; Wilcoxon rank-sum test: ∗p < 0.05, ∗∗p < 0.01, ∗∗∗p < 0.001.

Immunophenotypic analysis was performed on the surviving *ADA*^−/−^ mice treated with MND-ADA AAV8 and compared with age-matched untreated *ADA*^+/−^ and *ADA*^−/−^ mice on long-term ERT. The total numbers of splenocytes in all treatment groups were significantly lower compared with the numbers in untreated, immune-competent *ADA*^+/−^ mice, indicating immune reconstitution was incomplete in *ADA*^−/−^ mice treated with long-term ERT (p = 0.004) or with rAAV (p = 0.001) (Figure 2A). The total numbers of developing thymocytes (CD4^+^, CD8^+^, CD4^+^CD8^+^, and CD4^−^CD8^−^) after treatment with rAAV were similar to untreated *ADA*^+/−^ mice, and the numbers of CD8 and CD4^−^CD8^−^ were higher when compared with ADA^−/−^ on ERT (Figures 2A–E). However, the numbers of mature T and B lymphocytes in the spleen of *ADA*^−/−^ mice treated with rAAV (CD19^+^ p = 0.03, CD3^+^ p = 0.004, CD4^+^ p = 0.01, CD8^+^ p = 0.004) or with long-term ERT (CD19^+^ p = 0.02, CD3^+^ p = 0.002, CD4^+^ p = 0.001, CD8^+^ p = 0.001) were significantly lower compared with untreated, immune-competent *ADA*^+/−^ mice, suggesting that the lack of intracellular ADA expression with ERT and rAAV treatment may limit the extent of immune reconstitution. Lymphocyte functional studies were not performed.

Due to a move to a different vivarium and the required use of a sterile rederivation of the strain to avoid transfer of pathogens, the remainder of the studies described herein were performed on a sub-strain in which the FVB/129 of ADA KO mice were backcrossed for two generations to C57/Blk6 mice (FVB/129/C57BLK6). Additionally, only the MND-ADA rAAV was investigated because the EFS-ADA rAAV was less potent and associated with reduced survival.

To model older patients on long-term ERT prior to HSC GT, we maintained *ADA*^−/−^ mice on PEG-ADA ERT from birth and treated them with rAAV *in vivo* GT at 8–10 weeks of age with 1.0 × 10e12 gc/kg MND-ADA rAAV, and these mice comprise the adult group (adult). For comparison, litters of *ADA*^−/−^ and *ADA*^+/−^ mice were treated as neonates with 1.0 × 10e12 gc/kg MND-ADA rAAV8 and concomitant ERT for 30 days post-treatment, and these mice comprise the neonate group (neonate).

**Figure 3:**
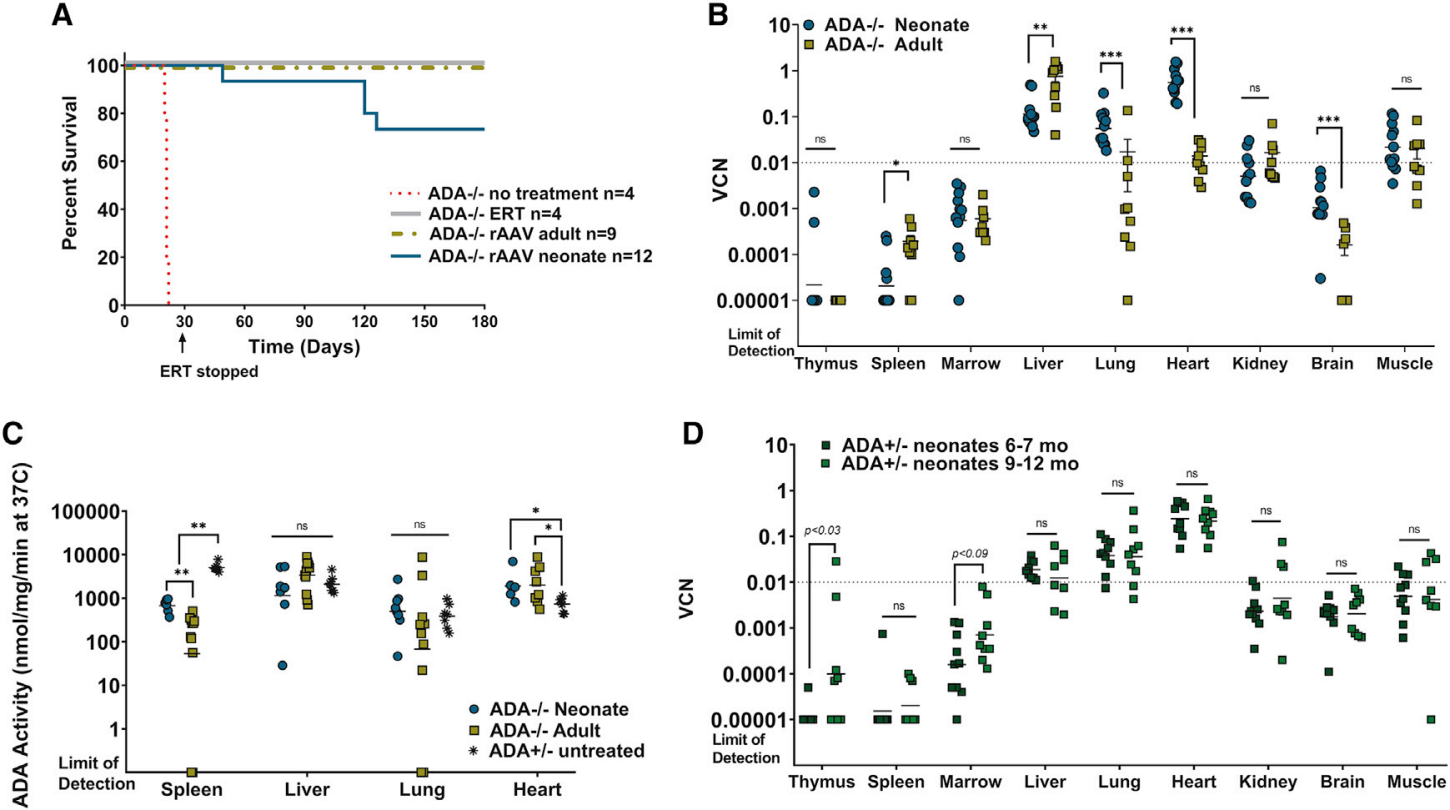
Survival, biodistribution, and ADA activity after rAAV *in vivo* GT in *ADA*^−/−^ neonates and adults (A) Survival after treatment with intravenous injection of rAAV *in vivo* GT; survival was tracked to day +180, at which time the animals were euthanized for analysis. Treated mice were compared with historic controls of untreated mice, which died by the third week of life. Survivorship was subjected to Kaplan-Meier analysis. (B) Tissue rAAV VCN; dotted line to indicate the putative threshold for survival in *ADA*^−/−^ mice as demonstrated in Figure 1. (C) Tissue ADA enzyme activity. (D) rAAV VCN in rAAV-treated *ADA*^+/−^ littermates out to 1 year. (B–D) Geometric means are plotted; Wilcoxon rank-sum test: ∗p < 0.05, ∗∗p < 0.01, ∗∗∗p < 0.001.

Survival was tested for 180 days in the neonate group and 90–110 days after treatment in the adult group. The probability of survival was significantly improved in all treated *ADA*^−/−^ mice compared with the historical control of untreated *ADA*^−/−^ mice (p < 0.001), regardless of age of treatment (Figure 3A). In the neonate group, the rAAV VCN was higher in heart (p < 0.001), lung (p = 0.0010), and brain (p = 0.001), but lower in liver (p < 0.001) and spleen (p = 0.01), when compared with the adult group (Figure 3B). ADA activity detected in the heart was significantly higher in the neonate group (p = 0.02) and in the adult group (p = 0.03) when compared with untreated *ADA*^+/−^ mice, whereas there was no difference in liver and lung activity in any group (Figure 3C). ADA activity in spleen was significantly lower in all rAAV-treated *ADA*^−/−^ mice when compared with untreated *ADA*^+/−^ mice (p = 0.001). To determine the persistence of rAAV in tissues after the initial growth period, we analyzed the rAAV-treated *ADA*^+/−^ littermates at 6–7 months or 9–12 months to monitor the biodistribution of rAAV sequences in tissues. There were no differences in tissue VCN in all tissue analyzed, indicating stable marking out to 1 year (Figure 3D).

In both studies, it was noted that *ADA*^−/−^ and *ADA*^+/−^ mice treated as neonates with rAAV often had an enlarged thymus upon gross inspection. However, microscopic examination of tissues revealed normal cellularity and normal thymic architecture (data not shown). Furthermore, flow cytometry of thymocytes stained with anti-CD4 and anti-CD8 revealed normal flow plots and subpopulation percentages. Interestingly, there was no evidence of thymic enlargement in the adult group in which the heart VCN was lower, but ADA activity was similar to the neonate group.

**Figure 4:**
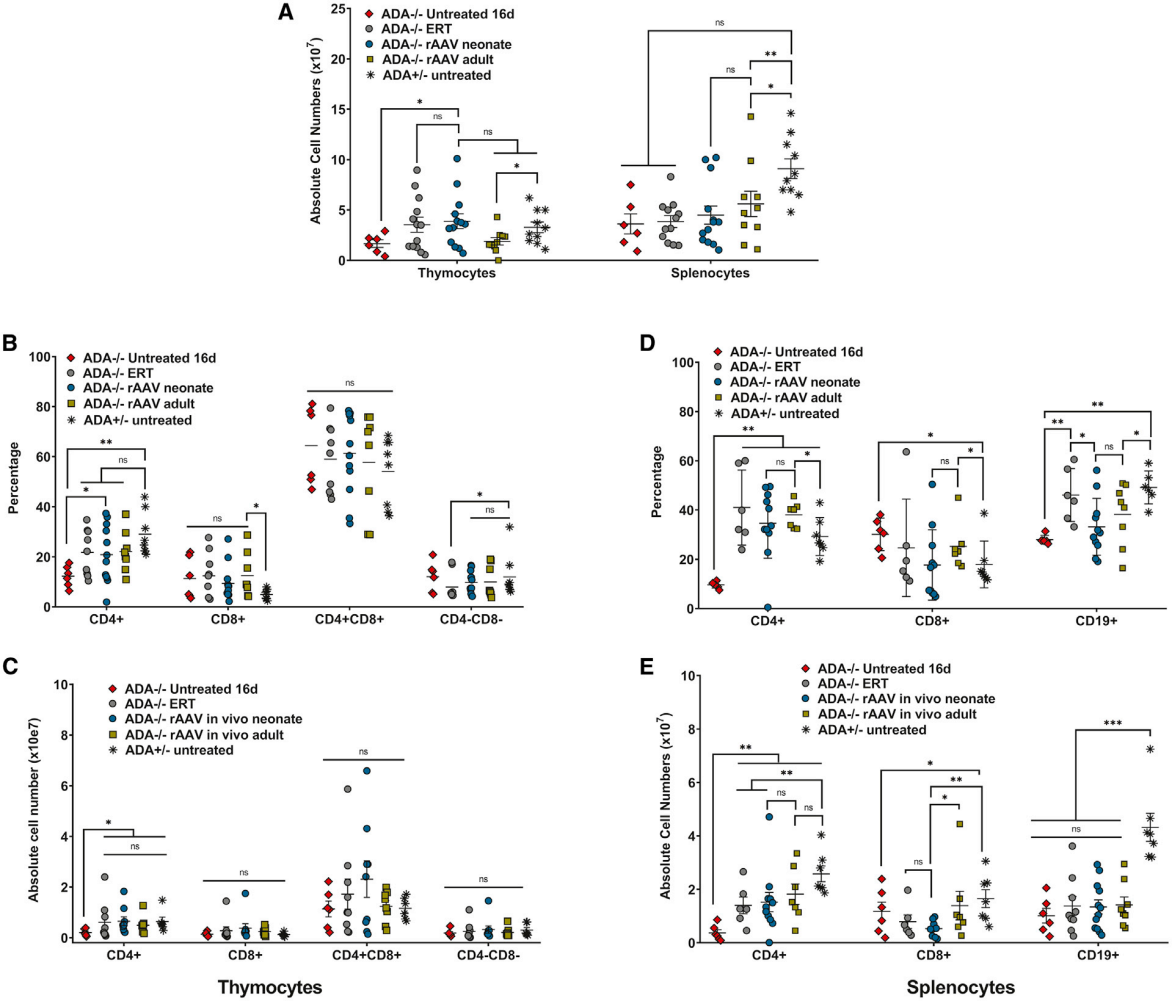
Immunophenotype and absolute cell counts after rAAV *in vivo* GT in *ADA*^−/−^ neonates and adults Comparisons were made with untreated *ADA*^−/−^ mice euthanized at 16 days of age prior to serious decline and death, as well as with age-matched *ADA*^−/−^ mice maintained on ERT and untreated *ADA*^+/−^ mice. (A) Total lymphoid organ cell counts. (B) Thymocyte subpopulations (percentage): single-positive CD4^+^, single-positive CD8^+^, double-positive CD4^+^CD8^+^, and double-negative CD4^−^CD8^−^. (C) Absolute thymocyte counts. (D) Splenocyte subpopulations: CD4^+^ helper T cells, CD8^+^ cytotoxic T cells, CD3^+^ T cells, and CD19^+^ B cells. (E) Absolute splenocyte counts. (A–E) Mean ± SEM are plotted; Wilcoxon rank-sum test: ∗p < 0.05, ∗∗p < 0.01, ∗∗∗p < 0.001.

Immunophenotypic analysis was performed on rAAV-treated *ADA*^−/−^ mice and compared with age-matched untreated *ADA*^+/−^ mice (*ADA*^+/−^) and *ADA*^−/−^ mice on long-term ERT (ERT), as well as with untreated *ADA*^−/−^ mice euthanized at P16. The numbers of thymocytes were reconstituted after treatment with rAAV *in vivo* GT at any age, but the numbers of splenocytes were reduced in all treated and untreated *ADA*^−/−^ mice compared with immune-competent *ADA*^+/−^ mice (untreated 16d, p = 0.01; ERT, p = 0001; neonate, p = 0.005; adult, p = 0.02) (Figure 4A). There was no difference in the numbers of thymocyte subpopulations isolated from treated *ADA*^−/−^ and untreated *ADA*^+/−^ mice (Figures 4B and 4C). However, the numbers of splenic CD19^+^ B cells were significantly lower in the treated and untreated *ADA*^−/−^ mice compared with the immune-competent *ADA*^+/−^ mice (untreated 16d, p = 0.001; ERT, p = 0001; neonate, p < 0.001; adult, p < 0.001), indicating incomplete B cell reconstitution with either long-term ERT or rAAV *in vivo* GT (Figures 4D and 4E). The neonate group had significantly lower numbers of splenic CD4^+^ (p = 0.01) and CD8^+^ (p = 0.001) cells compared with the immune-competent *ADA*^+/−^ mice, but there were no differences in splenic subpopulations in the adult group that had higher *ADA* gene marking in the spleen compared with the neonate group.

We hypothesized that treatment with systemic delivery of an rAAV expressing the human *ADA* transgene shortly after diagnosis would stabilize a patient to mitigate the metabolic consequences of ADA deficiency and create a more suitable environment for engraftment during a subsequent HSC treatment, such as allogeneic HSCT or HSC *ex vivo* GT. We reasoned rAAV would be also a good choice because of the low potential for integration and putative decrease in expression with growth and subsequent dilution of rAAV genomes.^17^ In this study, we set out to demonstrate the feasibility of a bimodal GT approach, which would entail stabilizing a neonate with rAAV as a source of enzyme followed by a subsequent LV-mediated HSC *ex vivo* GT as an adult.

Newborn *ADA*^−/−^ FVB/129/C57BLK6 mice (days 1–3) were treated by intravenous injection of 1.0 × 10e12 gc/kg of MND-ADA rAAV8 as neonates followed by transplantation at 2 months of age with *ADA*^−/−^ lineage-depleted bone marrow (lin^−^) cells genetically modified *ex vivo* with the EFS-ADA LV (bimodal GT; n = 8).^18^ Additional controls included *ADA*^−/−^ mice that were treated solely with one modality or the other: *ADA*^−/−^ neonates treated only with the rAAV (rAAV *in vivo* GT; n = 12) or 2-month-old *ADA*^−/−^ mice treated only with the *ADA*^−/−^ lin^−^ cells genetically modified *ex vivo* with the EFS-ADA LV (LV HSC *ex vivo* GT; n = 7).

**Figure 5:**
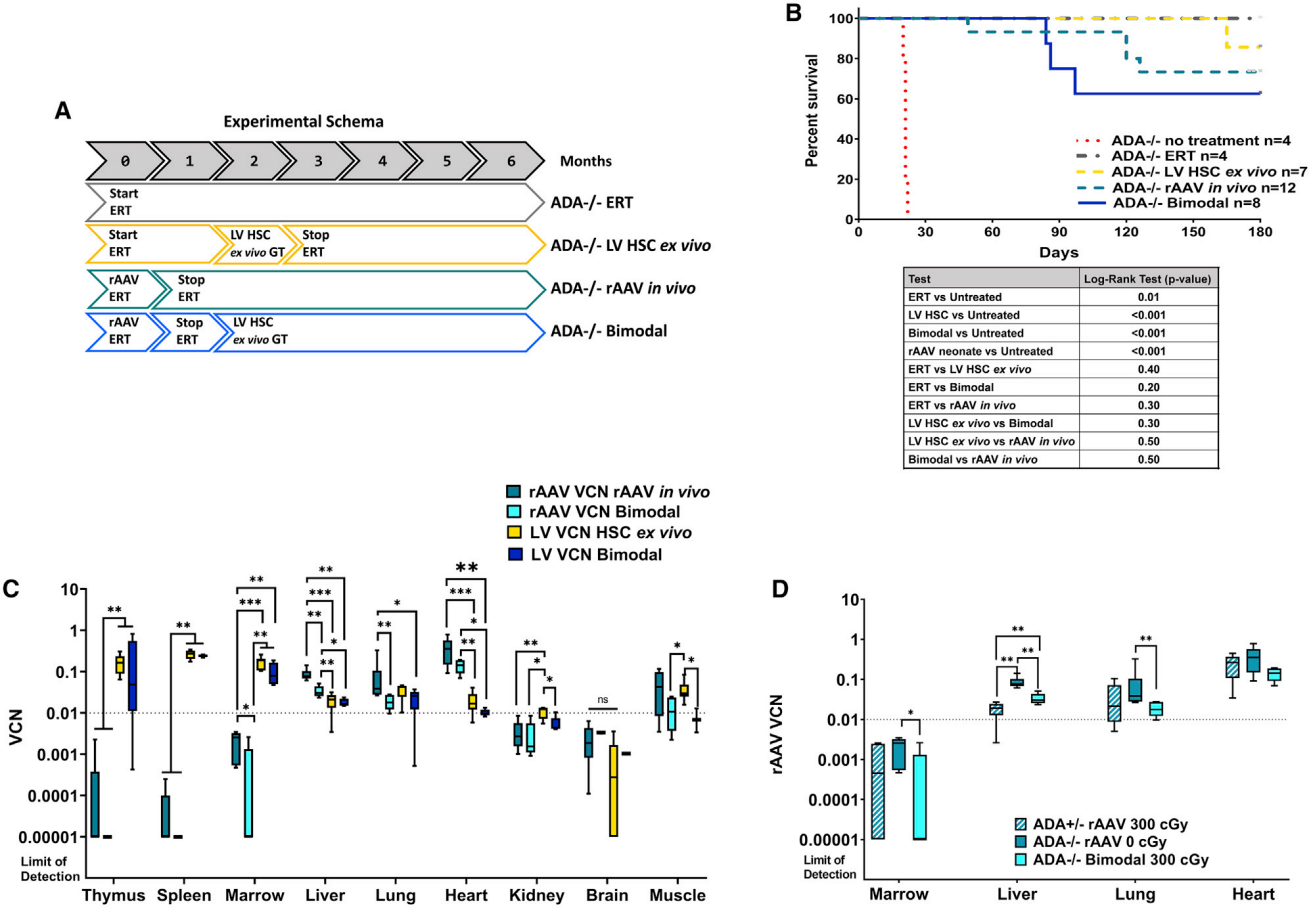
Survival and vector biodistribution after bimodal GT (A) Experimental schema: *ADA*^−/−^ mice were treated with rAAV *in vivo* GT as a sole treatment, with LV HSC *ex vivo* GT as a sole treatment, or with both as a bimodal GT treatment. (B) Survival was tracked to day +180, at which time the animals were euthanized for analysis. Treated mice were compared with historic controls of untreated mice that died by the third week of life. Survivorship was subjected to Kaplan-Meier analysis. All mice received ERT with PEG-ADA for 30 days after treatment with first gene therapy. (C) VCN was determined by qPCR using vector-specific primers/probes. Whisker plots with minimum and maximum are shown. (D) Tissue VCN in *ADA*^+/−^ mice treated with rAAV *in vivo* GT at post-natal days 1–3 with 300 cGy TBI (n = 6) and without TBI at 8 weeks (n = 10). Whisker plots with minimum and maximum are shown. Dotted line to indicate the putative threshold for survival in *ADA*^−/−^ mice as demonstrated in Figure 1. Wilcoxon rank-sum test: ∗p < 0.05, ∗∗p < 0.01, ∗∗∗p < 0.001.

The intent, if translated to the clinic, would be for rAAV to be the sole source of ERT. In order to test the ability of the ADA expressed from the rAAV to support individuals before and during the period of engraftment, *ADA*^−/−^ mice in the bimodal GT group did not receive exogenous ERT 30 days prior to the infusion of the LV-modified lin^−^ cells, nor was ERT supplied at any time after infusion (Figure 5A). However, as in the previous experiments, ERT was supplied for 30 days after the first GT administered to avoid large numbers of intercurrent deaths. All *ADA*^−/−^ mice treated with the genetically modified cells (LV HSC *ex vivo* GT and bimodal GT) were also conditioned with 300 cGy of total body irradiation (TBI) prior to infusion of the LV-modified cells. Conditioning is necessary for engraftment of genetically modified hematopoietic stem and progenitor cells (HSPC).^11^ As a control for the TBI, 2-month-old *ADA*^+/−^ congenic mice, similarly treated with rAAV as neonates, were also conditioned with 300 TBI on the same day as the 2-month-old mice *ADA*^−/−^ mice but were not transplanted (*ADA*^+/−^ rAAV 300 cGy). All surviving mice were euthanized at 6 months of age, and tissues were harvested for analysis.

The probability of survival was significantly increased in all *ADA*^−/−^ mice in the GT treatment arms compared with the untreated *ADA*^−/−^ historical control (p < 0.0001) (Figure 5B). There was no difference in survival in treated *ADA*^−/−^ mice. Survival with rAAV *in vivo* GT was 73.3%, LV HSC *ex vivo* GT was 85.7%, and bimodal GT was 62.5%.

VCN was determined by droplet digital polymerase chain reaction (ddPCR) using primers and probes specific for either the rAAV vector sequence (rAAV VCN) or the LV sequence (LV VCN); thus, the contribution of each vector was distinguishable. Overall, the rAAV VCN was significantly higher in liver and heart and significantly lower in thymus, spleen, and bone marrow (Figure 5C; Table 1). LV VCN in the bimodal GT group was not different from thymus, spleen, marrow, liver, lung, heart, and brain when compared with the LV HSC *ex vivo* GT group but was reduced in kidney (p = 0.02) and muscle (p = 0.02). In the bimodal GT group, which was conditioned with TBI, the rAAV VCN was reduced in marrow (p = 0.02), liver (p = 0.002), and lung (p = 0.01) when compared with the rAAV *in vivo* GT group, but when compared with the *ADA*^+/−^ rAAV group that was also conditioned with TBI, the rAAV VCN in marrow, liver, and lung was not different (Figure 5D). This finding suggests that the reduction of rAAV VCN in the bimodal GT group is due to the cytoreductive conditioning.

**Table 1:** Wilcoxon rank-sum test: VCN in group comparison (p values)

	rAAV *in vivo* compared with LV HSC *ex vivo*	rAAV *in vivo* compared with LV biomodal	LV HSC *ex vivo* compared with rAAV biomodal	rAAV *in vivo* compared with rAAV bimodel	LV HSC *ex vivo* compared with LV biomodal	rAAV bimodal compared with LV biomodal
Thymus	0.002^a^	0.01^a^	0.01^a^	0.30	0.54	0.01^a^
Spleen	0.002^a^	0.01^a^	0.01^a^	0.30	0.48	0.01^a^
Marrow	0.001^a^	0.004^a^	0.01^a^	0.02^a^	0.07	0.02^a^
Liver	0.001^a^	0.004^a^	0.01^a^	0.002^a^	0.48	0.03^a^
Lung	0.94	0.03^a^	0.10	0.01^a^	0.10	0.69
Heart	0.001^a^	0.002^a^	0.004^a^	0.09	0.08	0.01^a^
Kidney	0.005^a^	0.09	0.02^a^	0.52	0.02^a^	0.10
Brain	0.080	0.89	0.24	0.40	0.45	0.67
Muscle	0.750	0.13	0.02^a^	0.13	0.02^a^	0.79

a p values indicate significance.

**Figure 6:**
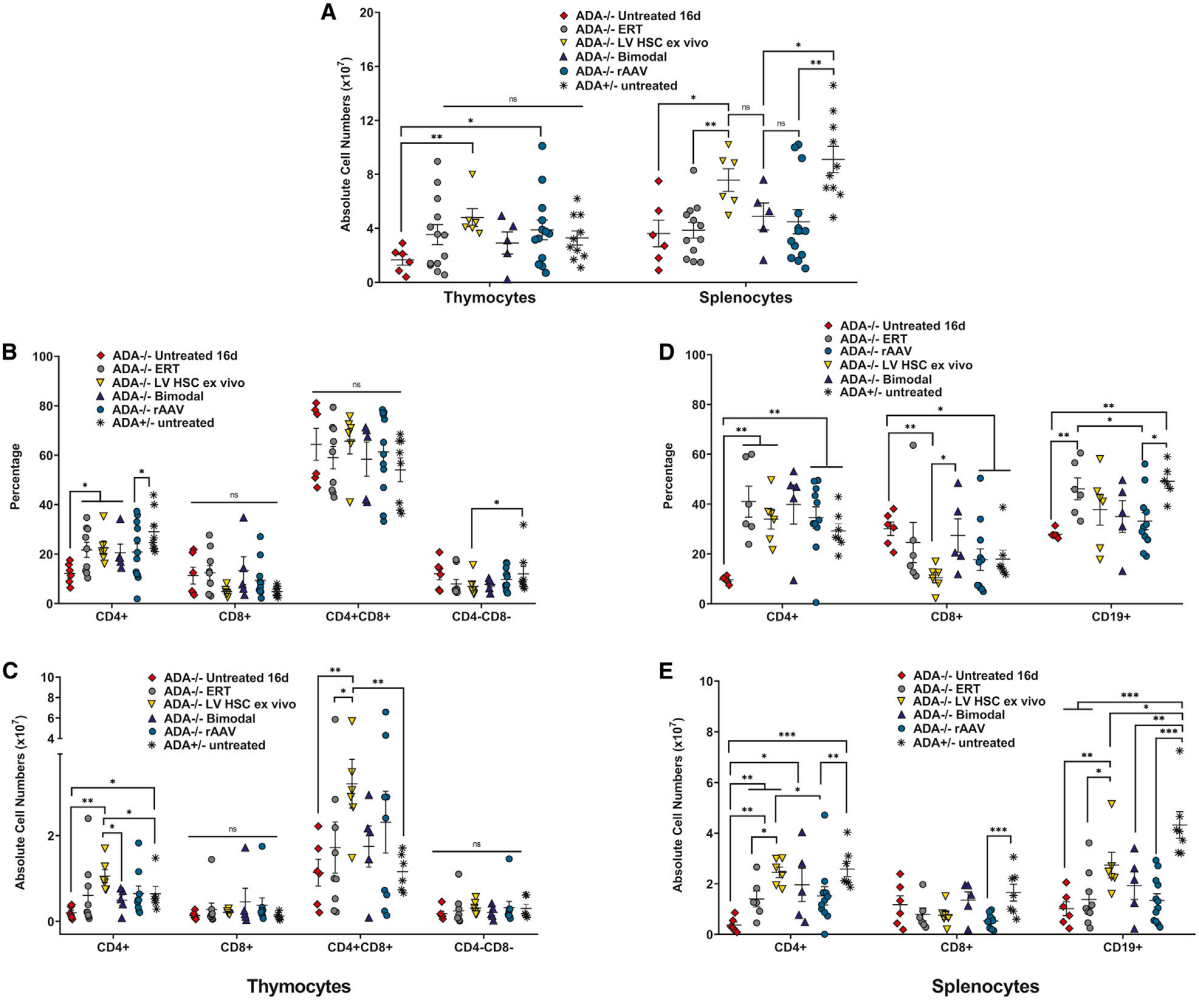
Immunophenotype and absolute cell counts after bimodal GT Comparisons were made with untreated *ADA*^−/−^ mice euthanized at 16 days of age prior to serious decline and death, as well as with age-matched *ADA*^−/−^ mice maintained on ERT and untreated *ADA*^+/−^ mice. (A) Total lymphoid organ cell counts. (B) Thymocyte subpopulations (percentage): single-positive CD4^+^, single-positive CD8^+^, double-positive CD4^+^CD8^+^, and double-negative CD4^−^CD8^−^. (C) Absolute thymocyte counts. (D) Splenocyte subpopulations: CD4^+^ helper T cells, CD8^+^ cytotoxic T cells, CD3^+^ T cells, and CD19^+^ B cells. (E) Absolute splenocyte counts. (A–E) Mean ± SEM are plotted; Wilcoxon rank-sum test: ∗p < 0.05, ∗∗p < 0.01, ∗∗∗p < 0.001.

As seen in the previous experiments, the rAAV GT group reconstituted thymocyte subpopulations and numbers, but not mature lymphocytes in the spleen when compared with untreated, immune-competent *ADA*^+/−^ mice, specifically splenic CD4^+^ cells (p = 0.008), CD8^+^ cells (p = 0.001), and CD19^+^ cells (p = 0.001) (Figures 6A–6E). In contrast, the LV HSC *ex vivo* GT group reconstituted both the thymocyte subpopulations and mature T cells in the spleen, but not the CD19^+^ cells (p = 0.035), when compared with untreated *ADA*^+/−^ mice. Immune cell reconstitution in the bimodal GT group was similar to the LV HSC *ex vivo* GT group with the exception of the CD4^+^ single-positive thymocyte population, which was significantly lower in the bimodal group (p = 0.017). These results suggest the ectopic ADA expression from the rAAV was not detrimental to mature lymphocyte survival in the periphery.

## Discussion

ADA deficiency is an inborn error of metabolism that causes SCID, as well as other system disorders, including pulmonary insufficiency, hepatic and renal dysfunction, skeletal abnormalities, and neurological manifestations, such as hearing loss, behavioral issues, and in some cases, cognitive deficits.^5^ Patients with ADA-deficient SCID are often diagnosed in infancy, and without treatment patients would die within the first years of life. Upon diagnosis, patients should be immediately treated with PEG-ADA ERT for stabilization and improvement of both immune function and pulmonary insufficiency.^5^ Typically, patients are eventually treated with a more definitive treatment, such as HSCT or HSC *ex vivo* GT, and ERT is used as a bridge therapy.^6^ In these studies, *ADA* KO mice were treated with rAAV *in vivo* GT as a sole treatment or as part of a bimodal GT approach in which young adult mice were treated with a LV-mediated HSC *ex vivo* GT after neonatal treatment with rAAV *in vivo* GT as a form of ERT.

It should be noted that the utility of rAAV8 was previously demonstrated in another study performed in a sub-strain of the ADA KO mouse model in which ADA was expressed in the placenta and foregut during gestation and in the foregut post-natally (JAX strain FVB;129-*Adatm1Mw Tg(PLFSADA)2465Rkmb/J*).^19^ This sub-strain has a normal lifespan, exhibits a partial immune deficiency, and did not allow for characterizing immune reconstitution and correction of the SCID phenotype after treatment with rAAV.

It is generally recognized that the mouse model used herein is very severe, and treatments must be robust to attain survival without ERT.^20^ In studies using this ADA-deficient mouse model, it was found that the accumulation of adenosine and deoxyadenosine led to the development of severe disturbances in immune cell development, rib cage alterations, renal abnormalities, and pulmonary insufficiency that could be ameliorated with the use of PEG-ADA ERT.^21^ A correlation was found between the amount of ectopic ADA supplied and the subsequent phenotypes observed. A low dose of PEG-ADA (100–300 U/kg/week intramuscularly [i.m.]) was found to be necessary for the amelioration of the pulmonary insufficiency without restoration of thymic development, and a higher dose was necessary to resolve the block in thymic development (1,000 U/kg intraperitoneally [i.p.]).^21^ The higher dosage resulted in ADA trough levels in plasma equivalent to a dose of 60 U/kg/week in ADA-deficient patients. In our experience, we find a dose of 300 U/kg/week i.m. provided for both the amelioration of the disease and resolution of the thymic block in development.^11^^,^^22^

In this regard, the *ADA*^−/−^ mice used in this study were maintained on PEG-ADA ERT for 1 month following the first application of a GT to avoid large numbers of intercurrent deaths before there was adequate vector ADA expression and to explore endpoints relevant to the human experience, such as immune reconstitution and survival without ERT after GT. To be clear, we do not expect that clinical development of an rAAV ADA GT would include concurrent treatment with PEG-ADA during the pre- or post-treatment period as it was used in this model.

rAAV8 was chosen, in part, due to its ability to cross blood vessel barriers and for the relatively low incidence of neutralizing antibodies in the general human population.^23^^,^^24^ In experiments using escalating doses of rAAV8, we observed that survival was vector dose dependent, but tissue marking was not vector dose dependent, and that all surviving mice had similar levels of marking in liver. In similarly treated *ADA*^+/−^ littermates who are not ADA deficient and have normal lifespans, there was a positive dose-dependent relationship between vector dose administered and VCN detected in both heart and liver, even at the lowest doses. These findings suggest there is a threshold for survival in *ADA*^−/−^ mice with these vectors. Non-pathogenic pulmonary insufficiency has been observed in ADA-deficient infants,^25^^,^^26^ but it is not as severe as the lethal phenotype in the mouse model, so it is not clear how relevant this threshold is for clinical benefit in human patients. Future studies should determine if there is potential for rAAV therapy to provide relief from skeletal, hepatic, renal, and neurological phenotypes.

In mice (*ADA*^−/−^ and *ADA*^+/−^) treated as neonates with high doses of rAAV as a sole treatment, we observed enlarged thymi that were associated with a high level of heart VCN. However, in *ADA*^−/−^ mice treated as adults, heart VCN was significantly reduced, and there was no incidence of enlarged thymi, indicating that rAAV treatment may be responsible rather than ADA deficiency itself. The high heart rAAV VCN was not surprising because cardiac muscle has been described as target tissue for rAAV8 in mice treated systemically as neonates;^23^ however, there was no mention of thymic enlargement in that particular model. The thymus, which lies anterior and superior to the heart, is often in direct contact to heart tissue, and it is possible the thymic tissue was dysregulated by the highly marked cardiac tissue and elevated ADA activity. With both thymocyte development and thymic architecture unaltered in mice treated as neonates or adults, the significance of this finding is unclear.

Another age-specific difference we observed was the reduced vector marking in the brain of mice treated as adults compared with mice treated as neonates. The blood-brain barrier (BBB) is not fully formed during the neonatal period and may have allowed for the higher level of rAAV8 transduction in the brain of neonates. In a previous study of LV *in vivo* GT by intravenous injection, brain VCN was 100- to 1,000-fold reduced in monkeys treated as neonates, compared with mice treated as neonates, suggesting the timing of BBB development may play a role.^16^ Another possible reason for increased brain VCN in mice treated during the neonatal period is that higher volumetric pressures are generated when a bolus of vector volume is injected intravenously. Nevertheless, expression of ectopic ADA in the brain may be relevant, because many patients experience neurological symptoms, including hearing loss, cognitive delay, and hyperactivity disorders associated with several MRI abnormalities, white matter alterations, and motor dysfunctions that are not corrected fully with ERT, HSCT, or HSC GT.^27^, ^28^, ^29^ Although rAAV8 resulted in limited brain marking, pre-clinical and clinical studies using rAAV9 have demonstrated transfer across the BBB,^30^^,^^31^ and perhaps additional studies with different serotypes are warranted.

Current HSC *ex vivo* GTs for ADA SCID couple PEG-ADA ERT to allow for systemic detoxification and stabilization prior to treatment and during the engraftment period.^11^^,^^19^ With that in mind, we investigated if rAAV *in vivo* GT could be coupled with a more definitive HSC therapy, months or years later. To model this bimodal GT approach, we treated ADA^−/−^ neonates with an intravenous injection of rAAV8 carrying the human ADA cDNA and 1-month ERT with PEG-ADA. At 2 months of age, 4 weeks after the last administration of ERT, the mice were treated with an LV HSC *ex vivo* GT without further administration of PEG-ADA. As an ERT, we expect that rAAV GT would initially result in slower detoxification rates when compared with PEG-ADA ERT, although this was not evaluated.

In theory, rAAV *in vivo* GT could be administered intravenously shortly after diagnosis. Detoxification and stabilization might take longer with rAAV compared with PEG-ADA ERT, but this intervention would be a single administration without concurrent ERT, necessitating a single shipment on dry ice, in a package that could be resupplied with dry ice during travel. This type of intervention could be lifesaving in the event regular refrigeration-temperature shipments (2°C–8°C) of PEG-ADA are not feasible due to geographical, political, and/or regulatory limitations.

Previously we found that systemic administration of an LV (LV *in vivo* GT) expressing human ADA corrected the murine model of ADA deficiency.^32^ Although LV *in vivo* GT has potential as an additional treatment option for older patients or patients with late onset of disease, the lack of HSC transduction suggested that re-administration might be necessary over a patient’s lifetime. Later studies revealed that in LV *in vivo* GT, similar to rAAV *in vivo* GT, re-administration was problematic due to strong inactivating immune responses after the first administration.^17^^,^^32^ Even though it would be highly unlikely because of extensive washing and *ex vivo* transduction, we wanted to eliminate any potential for developing neutralizing immune responses against the infused HSC transduced *ex vivo* with an LV. For this reason and because of the low amount of integration, we chose to use an rAAV rather an LV or integrase defective LV to stabilizable ADA^−/−^ mice at birth prior to receiving the LV HSC *ex vivo* GT.

Not surprising, in mice treated with the bimodal GT, LV VCN was 100- to 1,000-fold higher in the thymus, spleen, and marrow when compared with the rAAV VCN. Furthermore, the rAAV VCN in the bimodal GT group was reduced in bone marrow and lung when compared with unconditioned mice treated with only rAAV *in vivo* GT as neonates, but marking was not different when compared with similarly conditioned *ADA*^+/−^ littermates treated with rAAV *in vivo* GT as neonates. Taken together, these results suggest that in the bimodal GT mice, exposure to conditioning with TBI prior to the LV HSC *ex vivo* GT eliminated some of the rAAV transduced cells in the marrow, lung, and liver, and that the rAAV marked cells in the bone marrow were replaced with LV-modified HSCs to give rise to genetically marked progeny in the spleen and thymus. It has been hypothesized that extracellular ADA may blunt the selective survival advantage of corrected HSPC;^5^ however, this does not seem to be the case because LV marking was not reduced in marrow, thymus, or spleen compared with mice treated with only LV HSC *ex vivo* GT.

In bimodal GT, the transgene expression from the engrafted LV transduced HSCs would be expected to persist long term in rapidly dividing hematopoietic cells because LV readily integrates the transgene into the HSC genome and is passed on to blood cell progeny. In contrast, in rAAV-transduced cells, the transgene persists mostly as episomes, which are not duplicated with cell division and not passed on to progeny. For this reason, it is not clear how long rAAV will persist even in slowly dividing cells within post-mitotic tissues. In our study, liver VCN was significantly lower 6 months after treatment with rAAV *in vivo* GT in neonates compared with liver VCN 4 months after rAAV treatment as 2-month-old mice, suggesting that cell division during the growth phase diluted the rAAV. This observation was also made in other studies in which neonates were treated with rAAV and marking decreased over time because of liver growth post-natally.^17^ Although most rAAVs do not integrate into the transduced cell DNA, it is estimated that up to 1% of rAAV transductions result in rAAV integration,^33^ and long-term persistence of these integrated rAAVs is still relatively unknown. A recent study of dogs treated for hemophilia A with rAAV and followed for 8–10 years showed that the dogs with stable expression of Factor 8 have an accumulation of integrated rAAV in liver cells and have developed what appears to be dominant clones.^34^

In order to understand the theoretical scope of the potential utility and limitations of ERT, rAAV as a sole therapy treatment option, and the bimodal approach, we have constructed a table for comparison (Table 2). Included in the comparison are aspects related to treatment schedule, access, mechanism of action, and potential cost. There are many unknowns about some of these aspects, especially cost and market access, and based only on the very limited experience with GTs currently available in some markets.

**Table 2:** Theoretical utility and limitations of ERT, rAAV, and bimodal therapies

	ERT PEG-ADA	rAAV as sole therapy	rAAV in bimodal
Number of treatments	continuous; weekly or biweekly	once	once
Temperature shipment	4°C–10°C	−80°C	−80°C
Rapidity of action	within 24 h	unknown, but not greater than 30 days	unknown, but not greater than 30 days
Titration of response	yes, rapidly	no	no
Engraftment in subsequent definitive treatment	allogeneic HSCT unknown potential for increased risk of GvHD	N/A	allogeneic HSCT unknown potential for increased risk of GvHD
	HSC GT: none		HSC GT: none
Treatment access	can be difficult due to logistics of recurrent consistent delivery of temperature-sensitive enzyme	dry ice shipment to a treatment center	dry ice shipment to a treatment center
Market access	limited access	unknown	unknown
Cost	minimum of $500K/year (retail pricing of)	unknown; based on other rAAV GT $600K to $2M	unknown; based on other rAAV GT $600K to $1M; HSC *ex vivo* GT is unknown for United States (Strimvelis in United Kingdom at £660K); approval for two GT treatments may be difficult in some markets
Correction of neurological clinical benefit	no clinical benefit as unable to cross BBB	unknown in human; unlikely to cross BBB with this serotype	higher potential to have bone marrow HSC-derived monocytes cross BBB to contribute to microglia, but not formally investigated

K, thousand; M, million; N/A, not applicable.

In conclusion, rAAV *in vivo* GT has potential to be a lifesaving treatment, especially for those ADA-deficient patients for whom consistent delivery of ERT is not a viable option and also has potential as a bridge therapy to a subsequent, definitive HSC therapy in a bimodal GT approach. In this proof-of-concept study, we demonstrated that neonatal rAAV *in vivo* GT and subsequent LV HSC *ex vivo* GT, delivered as a bimodal GT, has potential as a treatment for ADA-deficient SCID. This bimodal approach may also be applicable to other inherited metabolic disorders where there is cross-correction by ectopic enzyme expression, such as in lysosomal storage disorders. However, this novel therapeutic approach would benefit from more long-term analysis in mice and other preclinical models to provide greater insight on its potential in humans.

## Materials and methods

### Experimental animals

Animal procedures and housing were in accordance with the Animal Research Committee and Division of Laboratory Animal Medicine and the National Institutes of Health guidelines at University of California, Los Angeles (UCLA) in the United States. All animals were handled in laminar flow hoods and housed in micro-insulator cages in pathogen-free colonies. Two related strains of the ADA KO mouse were used in these studies. Engineering of the two-stage KO has been previously described.^15^ The rAAV dose response study used the original strain of an ADA KO mouse with placental expression of an ADA minigene (JAX: FVB;129-*Adatm1Mw Tg(PLADA)4118Rkmb/J*, Stock No: 003265). The rAAV age study and the bimodal GT study used a partial backcross of the ADA KO mice onto the C57/Blk6 background.^18^^,^^22^^,^^35^ This sub-strain was created when the FVB/129 mice were backcrossed two generations to C57/Blk6 mice in 2001. The backcross was halted because after two generations, the mixed strain failed to produce *ADA*^−/−^ pups. Although the backcross strategy was abandoned, this partial backcrossed strain was kindly provided to the Kohn Laboratory in 2002 by Rodney Kellems and Michael Blackburn^36^ and re-derived at the Department of Laboratory Animal Medicine at UCLA in 2009. Despite the backcross, these mice have been used previously in transplant studies with no complications.^11^^,^^35^

Mixed litters of ADA^+/−^ and *ADA*^−/−^ mice were obtained by crossing *ADA*^+/−^ males with ADA^+/−^ females. Genotypes were determined by PCR following the PCR parameters of the genotyping protocol found on the Jackson Laboratory website. *ADA*^−/−^ mice were maintained by weekly i.m. injection of bovine PEG-ADA (300 U/kg/week i.m.) (Adagen; a kind gift from Leadiant Biosciences, Gaithersburg, MD, USA) until 30 days after the first application of a GT.

### Viral vector construction

The MND-ADA rAAV and EFS-ADA rAAV, comprised of a rAAV2 genome with an AAV8 serotype, were packaged by University of Pennsylvania Gene Therapy Program Vector Core. Quality control was executed via genome copy number titration, as described on the University of Pennsylvania vector core website (“Quality Control Core”). The MND-ADA rAAV vector titer was determined to be 4.83 × 10e12 gc/mL, and the EFS-ADA AAV vector titer was determined to be 2.4 × 10e12 gc/mL. The MND LTR U3 region is a gamma retroviral enhancer/promoter that drives expression of the human *ADA* cDNA in the MND-ADA rAAV.^37^ The human EFS drives expression of human *ADA* cDNA in the EFS-ADA rAAV.^38^

The EFS-ADA LV is a self-inactivating (SIN) LV based on the human HIV-1 virus.^39^ The SIN deletion in the 3′ long terminal repeat allows for self-inactivation of the viral enhancer and promoter after integration in transduced cells.^40^ The EFS promoter, described above, drives expression of codon-optimized human *ADA* cDNA. The EFS-ADA LV was made in the laboratory of Dr. Bobby Gaspar, University College London, Great Ormond Street Hospital and packaged in the Kohn laboratory as described by Carbonaro et al.^18^ Titer was determined on HT29 cells as described by Cooper et al.^41^

### Systemic administration of rAAV

Mouse experimental groups treated as neonates were injected via the facial vein with either MND-ADA rAAV or EFS-ADA rAAV at a dose of 1 × 10e10, 1 × 10e11, or 1 × 10e12 gc/kg in a volume of 50 μL using a technique previously described by Carbonaro Sarracino et al.^16^ Mouse experimental groups treated as adults were injected with MND-ADA rAAV at a dose of 1 × 10e12 gc/kg in a 100 μL volume into the tail vein.

### Lentiviral-mediated HSC *ex vivo* GT

Mice in the LV HSC *ex vivo* GT group and in the bimodal GT group were transplanted with lineage-depleted *ADA*^−/−^ bone marrow cells transduced *ex vivo* with the EFS-ADA LV. Donor *ADA*^−/−^ male mice were euthanized, and bone marrow was extracted from the femurs and humeral bones. To enrich for hematopoietic stem and progenitor cells, we lineage-depleted bone marrow cells using the Miltenyi MACs lineage depletion kit (Miltenyi Biotech, San Diego, CA, USA). Immunomagnetic depletion of lineage-positive cells was achieved with antibodies to differentiate blood cell populations conjugated to paramagnetic beads. The lin^−^ were plated at 0.5 × 10e6 cells/mL, pre-stimulated with activating cytokines for 20 h, and transduced with the EFS-ADA LV at a dose concentration of 3.3 × 10e7 TU/mL (MOI of 132) for 20 h, as previously described by Carbonaro et al.^18^ Recipient mice were irradiated with 300 cGy TBI with Cesium-137 before tail vein injection of the genetically modified lineage-depleted bone marrow cells resuspended in 0.9% normal saline in a volume of 100 μL.

### VCN determination

The thymus, spleen, heart, bone marrow, liver, skeletal muscle, kidney, and brain were all removed from the experimental or control animals upon death. DNA was extracted from these tissues using the DNeasy Blood and Tissue Kit (QIAGEN) according to the manufacturer’s protocol. VCN was determined by quantitative PCR (qPCR) with primers specific for human *ADA* cDNA (spanning exons 6 and 7) in the biodistribution study in *ADA*^−/−^ and *ADA*^+/−^ mice treated as neonates and adults. The primer sequences were 5′-GGT CCA TCC TGT GCT GCA T-3′ for the forward primer and 5′-CGG TCT GCT GCT GGT ACT TCT T-3′ for the reverse primer 5′-FAM-6-CCA GCC CAA CTG GTC CCC CAA G-TAMRA-3′.

To detect both vectors in mice treated in the bimodal study, we performed ddPCR. Primers and probes were designed to specifically detect either the rAAV ADA vectors or the HIV-1 based LV. To detect rAAV ADA vectors, the primers and probe were specific to sequences located in the 3′ end of the *ADA* cDNA into the leader region (forward primer: 5′-ATT TTT AAA AGA AAA GGG GG-3′, reverse primer: 5′-ACT CCA CCT TAA ACT TAT CRA AAA-3′; probe sequence 5′-FAM-6-ACG AAT TGC/ZEN/TTC CGA GTG AGA GAC AC/-IABkFQ’-3′). To detect the EFS-ADA LV, the primers and probe were specific for the HIV-1 Psi packaging signal sequences and were described by Cooper et al.^41^ The data were normalized to the uc378 gene locus copies, and these primer and probe sequences are also described by Cooper et al.^41^ Samples analyzed with both methods showed no difference in the VCN detected in tissue DNA (data not shown).

### ADA enzyme activity assay

ADA activity was measured with the Diazyme ADA Assay Kit (Diazyme, Poway, CA, USA). The kit contains a lyophilized calibrator, which was reconstituted for 30 min and stored in microcentrifuge tubes at −20°C. Standards for the assay were made by making serial dilutions of calibrator (51.4 U/L) in 0.1 M Tris buffer such that the dilution factors of Tris to calibrator are 0 (only Tris added), 0.125, 0.25, and 0.5. The undiluted calibrator was also included as a standard. Two lyophilized ADA controls with known ADA activity values were reconstituted and frozen at −20°C and used in the assay. Tissue samples were lysed with a tissue disrupter in a solution of 0.1 M Tris and 1:100 Halt Protease Inhibitor Cocktail (Thermo Fisher Scientific, Irwindale, CA, USA). Protein concentrations of the samples were determined by Bradford Protein Concentration Assay, and the lysed tissue sample was diluted to 5 μg/μL. These diluted samples were further serially diluted for final 1-, 3-, 6-, and 9-fold dilution factors in a solution of 0.1 M Tris buffer and protease inhibitor (1:100). The assay sample volume was 5 μL for each standard, control, or sample (neat and diluted) pipetted in duplicate into a 96-flat-well black plate. Using a multi-channel pipet, 180 μL of R1 was added to each well containing sample, standard, or control. The plate was incubated in a plate reader spectrophotometer (Tecan Infinity Series) for 3 min at 37°C, after which 90 μL of R2, the second reagent in the kit, was added in similar fashion as R1. The plate was incubated again in a plate reader for 5 min at 37°C. Immediately after 5 min, the plate was read at 550 nm to obtain a baseline reading, known as “Read 1.” Exactly 3 min later, a second measurement was taken (“Read 2”). Finally, 3 min after that, a third measurement was taken (“Read 3”). The ADA enzyme assay is linear for a range of 0–200 U/L, and the dilutions or neat sample readings that fell within range were averaged and taken as the valid reading. The ADA enzyme assay gives values in units per liter (U/L), which were later converted and reported as units per minute per 1 × 10e8 cells (the amount of ADA needed to convert 1 nmol adenosine to inosine per minute per 1 × 10e8 cells at 37°C).

### Immunophenotypic analysis

Total T cell counts (CD3^+^, CD4^+^, and CD8^+^) and B cell counts (CD19^+^) were determined by flow cytometry, as previously described by Carbonaro et al.^35^ In brief, cell suspensions were made from the large lobe of the thymus and apical half of the spleen with mechanical pressure through a 70-μm screen. Red cells were lysed, and the cells were counted and stained as previously described. Flow cytometric analysis was performed to determine the immunophenotypic tissue subpopulations. Total organ cell counts and absolute cell counts were normalized mathematically by multiplying the total organ cell count by the proportion of cells that were a specific immunophenotype.

### Statistical analysis

Descriptive statistics, such as mean and standard error, were reported and presented graphically for quantitative measurements. Normality assumption was checked for outcomes before statistical testing. Wilcoxon rank-sum tests were performed for between-group comparisons on quantitative measures. Spearmen correlation was used to describe the correlation between vector marking and tissue ADA enzyme activity. Kaplan-Meier estimates of mice survival were calculated and presented in figures. Log rank test was performed to compare survival among groups over time. An alpha (type I error) of 0.05 is used as the cutoff for significance for two-sided statistical tests. All statistical analyses were carried out using statistical software SAS version 9.4.^42^
